# Complement System as a Target for Therapies to Control Liver Regeneration/Damage in Acute Liver Failure Induced by Viral Hepatitis

**DOI:** 10.1155/2018/3917032

**Published:** 2018-10-08

**Authors:** Juliana Gil Melgaço, Carlos Eduardo Veloso, Lúcio Filgueiras Pacheco-Moreira, Claudia Lamarca Vitral, Marcelo Alves Pinto

**Affiliations:** ^1^Laboratório de Desenvolvimento Tecnológico em Virologia, Instituto Oswaldo Cruz, Fundação Oswaldo Cruz, Rio de Janeiro, Brazil; ^2^Departamento de Patologia, Instituto Nacional do Câncer, Rio de Janeiro, Brazil; ^3^Hospital Federal de Bonsucesso, Rio de Janeiro, Brazil; ^4^Departamento de Microbiologia e Parasitologia, Instituto Biomédico, Universidade Federal Fluminense, Niterói, Brazil

## Abstract

The complement system plays an important role in innate immunity inducing liver diseases as well as signaling immune cell activation in local inflammation regulating immunomodulatory effects such as liver damage and/or liver regeneration. Our aim is to evaluate the role of complement components in acute liver failure (ALF) caused by viral hepatitis, involving virus-induced ALF in human subjects using peripheral blood, samples of liver tissues, and ex vivo assays. Our findings displayed low levels of C3a in plasma samples with high frequency of C3a, C5a, and C5b/9 deposition in liver parenchyma. Meanwhile, laboratory assays using HepG2 (hepatocyte cell line) showed susceptibility to plasma samples from ALF patients impairing *in vitro* cell proliferation and an increase in apoptotic events submitting plasma samples to heat inactivation. In summary, our data suggest that the complement system may be involved in liver dysfunction in viral-induced acute liver failure cases using ex vivo assays. In extension to our findings, we provide insights into future studies using animal models for viral-induced ALF, as well as other associated soluble components, which need further investigation.

## 1. Introduction

Acute liver failure (ALF) is a complex and rare clinical syndrome characterized by the development of severe liver dysfunction, promoted by extensive death of functional cells. The mortality rate is close to 80%, and liver transplantation [1–4] is the only treatment modality available to this date. ALF can be induced by viral infections and toxicity promoted by drug abuse or alcoholism. In Brazil, viral infections are responsible for the majority of ALF cases, including viral hepatitis viruses such as hepatitis A virus (HAV) and hepatitis B virus (HBV), as well as coinfections related to hepatitis C virus (HCV), HIV, Epstein-Barr virus, and CMV [5–7].

Inflammatory response is strongly associated with liver diseases, and activation can trigger liver damage with aggressive hepatocyte loss [8]. In ALF, extensive liver injury can be caused by a variety of molecules of the innate immune system, and the complement system plays an important role in this process [9, 10]. The complement system is the major component of innate immunity; its protective function starts with a cascade of proteases and soluble factors that activates the immune system, stimulating microbial clearance [11]. Nevertheless, the function of the complement system is associated with processes of development, degeneration, and regeneration of multiple organs [12–14]. Anaphylatoxins C3a and C5a present potential chemoattractant activity to amplify immune cell recruitment such as macrophages and neutrophils into the liver parenchyma [15]; quickly, C5a is converted to C5aDesArg *in vivo*, leading to local inflammation [16]. Following the complement cascade, C5b with C6–C9 forms the “membrane attack complex” (MAC), capable of lysing infected cells and pathogens [17, 18].

A susceptible cell culture system and animal models are important to improve knowledge of viral liver pathogenesis and promising therapies. However, useful viral-induced ALF animal models are not simple; in addition, the diversity of liver cell lines is used to study therapy interventions and also to understand mechanisms related to liver diseases, such as viral hepatitis [19, 20]. Here, we assessed the toxicological effects of the complement system in the peripheral blood of patients during acute liver failure.

## 2. Materials and Methods

### 2.1. Study Population and Samples

Blood samples (*n* = 8) and liver samples (*n* = 6) from ALF patients and blood samples (*n* = 10) and liver samples (*n* = 1) from healthy subjects (HS) were collected at the Liver Clinic/Hospital Federal de Bonsucesso, Rio de Janeiro, Brazil, between February 2004 and November 2013.

Inclusion criteria included the presence of coagulopathy (INR ≥ 1.5) and encephalopathy score above II, according to O'Grady et al. [21] and as described previously [7, 22, 23]. Clinical details of the study population are shown in Table 1.

Screening to investigate the etiological agent involved in ALF cases as well as in samples of healthy donors was performed for HAV, HBV, and HCV detection and also for other infectious and autoimmune disorders as described by Melgaço et al. [7].

The adopted protocol performed complied with relevant laws and institutional guidelines according to the ethical standards of the Declaration of Helsinki approved by the Institutional Review Board of Fiocruz (#222/03).

### 2.2. Laboratory Assays

#### 2.2.1. Detection of C3a Levels in Peripheral Blood Circulation

Blood samples (9 mL) were centrifuged (200 × *g*/10 min) to separate plasma from other products. Plasma samples were aliquoted and stored at −70°C until laboratory assays. Initially, plasma samples were divided into two groups: in the first one, plasma was thawed at room temperature (RT) overnight and in the second one, plasma was thawed at RT overnight and submitted to temperature complement inactivation by heating at 57°C for 30 min. To detect C3a levels in plasma samples, a commercial kit was used according to the manufacturer's instructions (eBioscience, cat #BMS2089TEN, San Diego, CA, USA).

#### 2.2.2. Detection of Anaphylatoxins and Membrane Attack Complex (MAC) in Liver Tissue Samples

Liver samples were obtained at the time of transplantation and stored in liquid nitrogen until immunofluorescence assays, sectioned at 5 *μ*m, and stained with antibodies to membrane attack complex C5b/9 (clone: aE11, Santa Cruz cat #SC-58935, Brazil); the C3a component (clone: K13/16, Santa Cruz cat #SC-47688, Brazil) and C5a component (clone: 2952, Santa Cruz cat #SC-52634, Brazil) were applied to the sections at a dilution of 1 : 50 for 1 hour at 37°C. After incubation, sections were washed in phosphate-buffered saline (pH = 7.2) and incubated with the secondary antibody Alexia Fluor 488^®^ (Abcam cat #AB-150113, USA) at a dilution of 1 : 1000. Photomicrographs were taken in a confocal microscope FV10i-O (Olympus, Japan) using FV10-ASW software. Calculation of marked areas was carried out using the software ImageJ (https://imagej.net/ImageJ).

#### 2.2.3. *In Vitro* Evaluation of ALF Soluble Components

Hepatocellular carcinoma lineage (HepG2, ATCC® number: HB-8065™) was chosen to evaluate ex vivo effects of ALF soluble components from plasma samples, since they are a well-established model for hepatocyte metabolism that under specific culture conditions maintains normal cell functions [24, 25]. HepG2 cultures were maintained in RPMI1640 (pH 7.4) (Gibco, USA) supplemented with 10% fetal bovine serum (FBS) (Gibco, USA) and 2 mM L-glutamine (Merck, Germany) in 75 cm^2^ bottles at 37°C in a CO_2_ humidified incubator. The medium was changed twice weekly, and passages were performed using trypsinization solution (0.2% and 0.02% versene in RPMI1640 medium). HepG2 confluency was regularly checked. The plasma of healthy subjects and ALF plasma samples were added to HepG2 cultures to assess the hypothesis of their toxicological effects. HepG2 cultures in a confluent monolayer were kept for 24 hours at 37°C in 24-well plates (5 × 10^4^cells/well). Then, different concentrations of plasma samples (complement heat-inactivated and noninactivated) diluted in RPMI1640 (0.1%, 1%, and 10%) were added to HepG2 cultures and incubated for 48 hours. Then, to assess viability, HepG2 cultures were labeled using 5 *μ*M of fluorescent vital dye cell trace in RPMI1640 (CFSE-FITC, Invitrogen®, USA). Negative proliferation percentage using CFSE-FITC was quantified by flow cytometry. Positive control of the absence of cell proliferation was carried out using colchicine at 10 *μ*M [26]. Negative control was performed adding RPMI1640 with 10% fetal bovine serum (FBS) heat-inactivated (57°C, 30 min) in HepG2 culture.

Levels of liver enzymes (ALT and AST) in supernatant of cell cultures were quantified to assess cellular damage and apoptosis caused by ALF plasma samples (Diasys®, Germany); HAV and HBV viral load quantification was performed by real-time PCR [27, 28]. HepG2 cells were incubated with *α*-CD95-FITC (annexin V, clone DX2, BD Pharmingen, USA). In this assay, HepG2 cells were also kept for 48 h, and positive control was assessed using ascorbic acid (AA) (70 *μ*M) for a 48 h incubation [29]. Blockage of membrane pores was achieved using specific phosphate saline solution (5% of 0.1 M BSA) and not fixed, according to the previous description [30].

After incubation, cells were analyzed by flow cytometry in both assays, 20,000 live cells were analyzed using a FACSCalibur™ flow cytometer with four fluorescence channels (FL1-530/30, FL2-585/42, FL3-670LP, and FL4-661/16), and off-line analysis was performed using FlowJo software (version 10.0.5) (FlowJo, LLC data analysis software, USA).

#### 2.2.4. Statistical Analysis

Statistical analyses were performed using Kruskal-Wallis and Dunn's multiple comparison test. ^∗^*p* ≤ 0.05, ^∗∗^*p* ≤ 0.01, and ^∗∗∗^*p* ≤ 0.001. All statistical analyses were performed using GraphPad Prism software version 7.

## 3. Results

### 3.1. The Lowest C3a Levels Detected in ALF Plasma Samples

**Figure 1:**
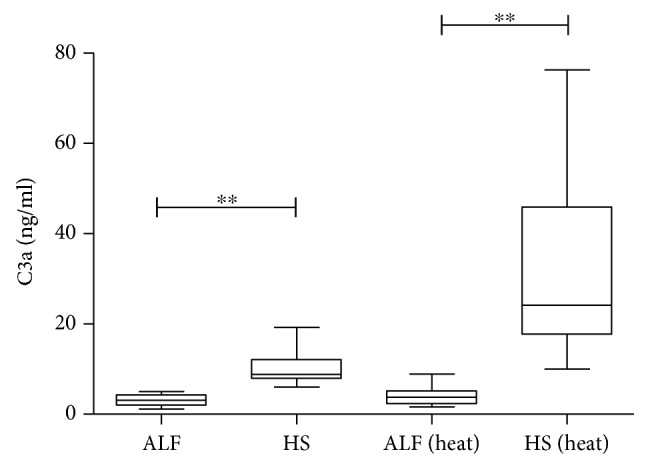
Soluble component C3a of complement system characterization of plasma samples from acute liver failure patients (ALF) and healthy subjects (HS) under heat inactivation (heat) and without heat inactivation.

Plasma samples from ALF patients and healthy subjects were submitted to C3a detection assays. Comparatively, Figure 1 shows superior levels of C3a detected among healthy donors (both heat-inactivated and noninactivated samples) even after thermal treatment (*p* = 0.0419).

### 3.2. Complement System Was Detected in Liver Samples from ALF Patients

**Figure 2:**
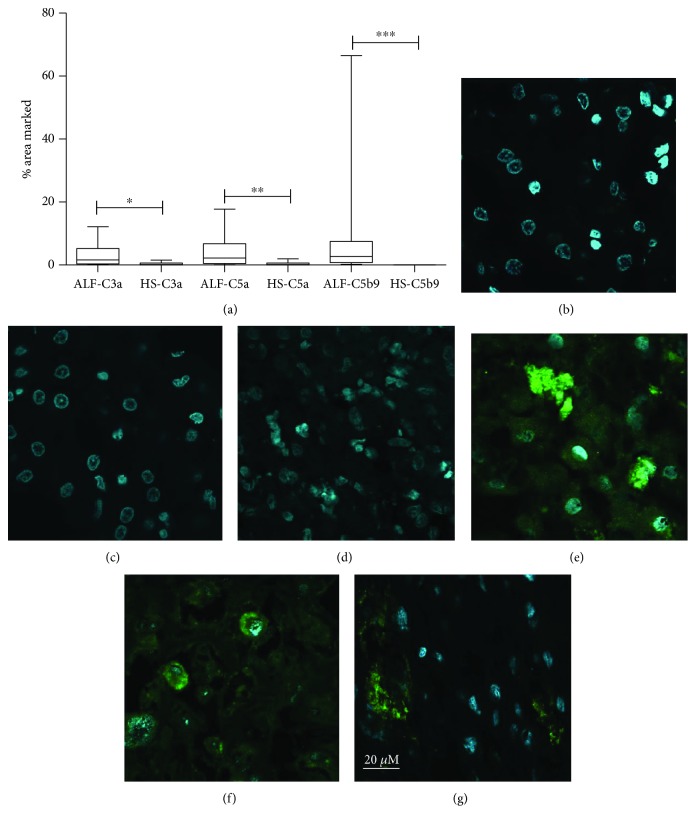
Liver deposit of anaphylatoxins C3a, C5a, and C5b/9 (MAC): (a) percentage of the area marked with anaphylatoxins in healthy subjects (HS) and acute liver failure (ALF) samples of liver tissue; (b–g) immunofluorescence assay (IFA) of liver samples tested for *α*C3a (b, e), *α*C5a (c, f), and *α*C5b/9 (d, g); the yellow underlined figures are from healthy subjects (b, c, d); the red underlined figures are from acute liver failure patients (e, f, g). Confocal microscopy zoom was of 400-fold.

Comparatively, a significant high percentage of C3a, C5a, and C5b/9 in labeled liver cells from ALF patients (Figure 2) was detected in tissue sections.

### 3.3. ALF Plasma Samples Induce Antiproliferative Activity and Apoptosis of *In Vitro* HepG2 Expansion

**Figure 3:**
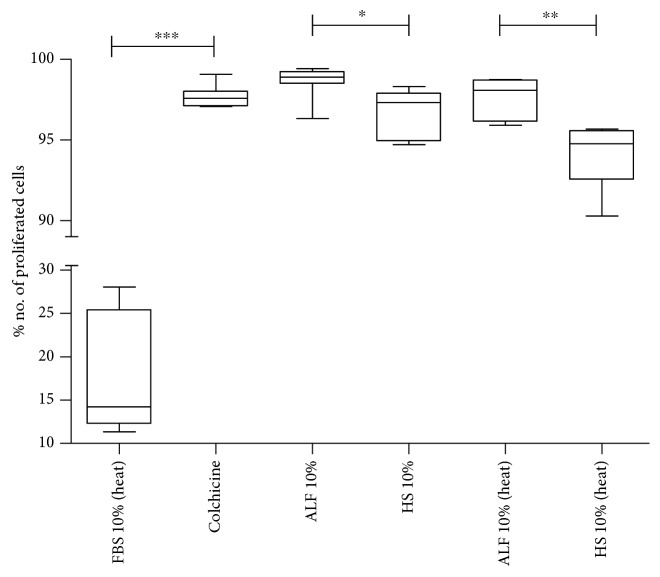
Effect of plasma (10%) from acute liver failure patients (ALF) and healthy subjects (HS) on HepG2 proliferation. Abbreviations: heat: heat-inactivated; ALF 10% vs. ALF 10% (heat), *p* = 0.0489.

Impairment of *in vitro* HepG2 expansion in the presence of 10% heat-inactivated (EC50, *p* = 0.0034) and noninactivated ALF plasma samples (EC50, *p* = 0.0153) was observed despite reduced circulation of C3a in ALF patients, similar to what was detected in positive controls (colchicine) (Figure 3). Results are presented in a 10% concentration of plasma samples diluted in RPMI1640 media.

**Figure 4:**
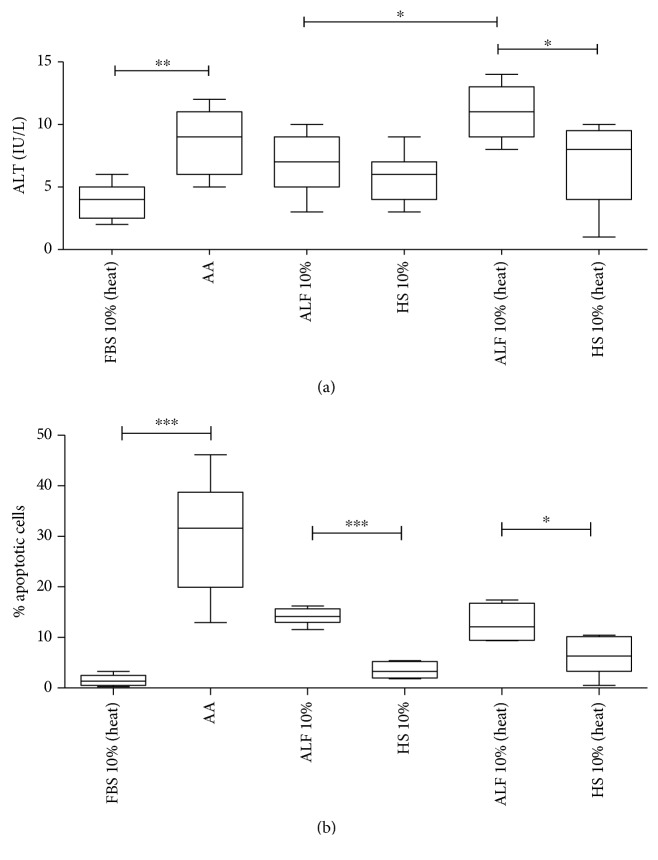
Effect of plasma (10%) from acute liver failure patients (ALF) and healthy subjects (HS) on a HepG2 cell line. (a) Measurement of *in vitro* alanine aminotransferase (ALT): FBS 10% (heat) vs. ALF 10%, *p* = 0.0265; FBS 10% (heat) vs. ALF 10% (heat), *p* = 0.0014. (b) Apoptosis evaluation. Abbreviations: FBS: fetal bovine serum; AA: ascorbic acid; heat: heat-inactivated.

To evaluate membrane damage in HepG2 cultures, alanine and aspartate aminotransferase levels (ALT and AST) in supernatant after ALF plasma exposure were measured and no changes in AST levels were found (data not shown). ALT levels were significantly higher in heat-inactivated ALF plasma than in plasma of healthy subjects and noninactivated ALF plasma, as displayed in Figure 4(a). In addition, we also explored the viral load of hepatitis A virus (HAV) and hepatitis B virus (HBV) in plasma samples used in cell cultures before and after thermal inactivation and on supernatant after ALF plasma exposure. No significant changes in viral load were found before and after thermal inactivation (*p* > 0.05), as well as after HepG2 exposure to ALF plasma samples (*p* > 0.05) (Table 2).

Subsequently, by extending our analysis to annexin V, the median percentage of apoptotic cells expressing annexin V was significantly high in the presence of 10% heat- and noninactivated ALF plasma samples compared with that of HS samples (Figure 4(b)).

## 4. Discussion

The complement system activation occurs in viral liver diseases [31–35]. Despite preventive measures, some viral liver diseases are widespread in developing countries causing acute hepatitis and its worst outcome is acute liver failure [33, 36]. Here, we confirmed the early impact of ALF plasma samples on HepG2 proliferation and viability, mimicking ALF liver environment. Some studies established the effects of the complement system on liver regeneration; however, the effective involvement of the components in viral liver injury and regeneration is still unknown [14, 18].

Our findings displayed a striking loss of C3 levels in peripheral blood from ALF patients, followed by high deposition of complement system components, such as C3a, C5a, and C5b-9 (MAC) in the liver parenchyma (liver explant) at the time of transplantation. C3 is a major component of the complement cascade, present at the early stage of liver inflammation [37], and C3 can regulate efflux and metabolism of steroid lipids in hepatocyte proliferation [18, 38]. In our previous report, plasmatic elevation titers of systemic inflammatory mediators (TNF-*α*, IL-6, IL-8, IL-10, IFN*γ*, and total mtDNA) were demonstrated in ALF patients [7, 22, 23, 39]. Similarly, other authors described the synergic effect of IL-1 beta and IL-6 on C3 mRNA transcription and C3 secretion in rat hepatocytes [40] since hepatocytes and Kupffer cells constitutively express receptors for C3 and C5a [40, 41].

In this study, viral-induced ALF patients with reduced C3a levels were associated with critical and progressive clinical conditions (donor shortage) and liver dysfunction. Similarly, nonviral etiologies [42, 43], LPS/D-GaIN-induced ALF [9], and chronic hepatitis cases [44, 45] could be justified by protein aggregates mediated by vitronectin or clusterin (complement regulatory proteins) [46], limited to the measurement of the complement components [45]. Displacement of the complement system from peripheral to central compartments in viral-induced ALF patients was already described in ALF animal models [15, 18] which were not exactly similar between animal and human studies [47]. Additionally, this study highlights that reduced levels of C3a in plasma from viral-induced ALF patients could not stimulate division of healthy hepatocytes since C3a biosynthesis depends on the normal liver function [32] as discussed by other authors in mouse models [15]. Hepatic deposition of components found in higher percentage in ALF liver samples could also suggest attempts for liver regeneration [18, 38, 48], which were not successful [10]. Despite the decrease in C3a levels in the plasma, its function cannot be ignored since it can trigger several cellular communications with different functionalities [13, 49]. On the other hand, lower C3a plasma levels and liver deposition can be a signal of liver “immunological storm” inducing hepatic damage [9, 32, 44, 45]. Those contradictory aspects discussed here raise the need for further investigation to understand the immune system in ALF syndrome.

Surprisingly, we observed a significant increase in C3a levels when plasma samples from healthy subjects were heat-inactivated. Contradictorily, some studies demonstrated that heat may influence [50] or not [51] immunoassay results, also associated with antibodies' interaction with protein activity after heating [50–52]. In our protocol, heat inactivation was performed at 57°C for 30 minutes; some studies described that complete inactivation of complement components in human serum can be effective for 30–60 minutes at 57°C [52, 53]. However, Moore and colleagues showed that C3b remained detectable and functional in decreased levels after heat inactivation of human sera [52].

Relevant weaknesses were identified in this study such as (i) soluble plasma components (endotoxin, hormones, bile acid, and other proteins); (ii) the reduced number of patients enrolled in this study related to the reduced number of liver transplants now occurring in Rio de Janeiro, Brazil; and (iii) scarce cases of ALF syndrome (0.5–1% of acute hepatitis cases [5, 7]).

In addition, we observed that *in vitro* HepG2 proliferation exposed to heat- or noninactivated plasma samples from ALF patients was significantly reduced, confirming our hypothesis related to the impairment of replicative capacity of resident hepatocytes. Intriguingly, during the early stages of liver damage, inflammatory cytokines induce healthy hepatocyte division [54]; however, during liver failure, its regenerative process is inadequate to match rapid, confluent loss of hepatocyte mass and function [8, 10].

Impairment of *in vitro* cell expansion could trigger the apoptotic process with massive loss of liver cells in ALF leading to interruption of liver regeneration, as observed in our findings (Figures 3 and 4) and described by others [43, 55, 56]. Other studies also showed plasma effects on hepatocyte cell lines affecting metabolism [57, 58]. In addition, heat inactivation may not be enough to abrogate the function of the complement system in ALF [52]. Also, biochemical thermal alterations in the complement and other components together can trigger an apoptotic event and/or may affect HepG2 cell proliferation [13, 49, 57, 58].

The viral load is another relevant variable that affects HepG2 proliferation. No significant changes were found in the viral load before and after ALF exposure. After heat inactivation, a slight decrease in the viral load was observed (Table 2), as described by others [59]. Probably, viable particles were not present in those samples, since plasma samples with no more than 4 log_10_ copies/mL were selected for this study, and HepG2 cell lines were not susceptible to HBV or HAV replication [60–62].

Our results raise the possibility of C3 as a target for new therapeutic approaches based on C3 function in hepatocytes for liver regeneration attempts. Although we cannot predict possible influence efficacy in this type of therapy, the knowledge of C3 effects on immortalized human hepatocyte cell lines could be useful to assess hepatocyte regeneration. It is noteworthy that other components (phosphate, creatinine, bile acid, and reactive oxygen species, among others) need further investigation since soluble factors are associated with fulminant hepatitis outcome [63–66]. However, we are studying an *in vitro* system using human samples to assess the role of primary complement component (C3a) in liver environment simulation.

Finally, lower levels of complement C3 in viral-induced ALF followed by a high frequency of C3a, C5a, and C5b/9 deposition in the dysfunctional liver parenchyma combined with higher HepG2 susceptibility suggest that therapies targeting the complement pathway should be further investigated to control liver regeneration/damage in fulminant hepatitis caused by viral hepatitis. Animal models should be the next very useful step to monitor ALF therapy since knockout studies using anticomplement antibodies, small interfering RNAs, and recombinant complement protein may be useful to evaluate the function of complements on cell proliferation and apoptosis.

## Figures and Tables

**Table 1:** Characteristics of the study population.

Subjects	Samples	Age	Gender	ALT	AST	INR	Encephalopathy	Outcome	Diagnostic	Etiology
1	HS1	29	F	14	21	1	None	NA	Healthy	None
2	HS2	27	M	16	22	1	None	NA	Healthy	None
3	HS3	29	F	13	19	1.1	None	NA	Healthy	None
4	HS4	26	M	23	33	0.9	None	NA	Healthy	None
5	HS5	46	F	18	26	0.8	None	NA	Healthy	None
6	HS6	31	F	11	15	0.9	None	NA	Healthy	None
7	HS9	26	F	16	25	0.9	None	NA	Healthy	None
8	HS10	33	F	13	18	1.1	None	NA	Healthy	None
9	ALF1	5	F	326	331	6.7	IV	Survival	Acute liver failure	HAV
10	ALF2	14	M	877	1790	4.32	IV	Death	Acute liver failure	HAV
11	ALF3	7	M	1562	5611	2.55	III	Death	Acute liver failure	HAV
12	ALF4	14	M	313	330	8.64	IV	Death	Acute liver failure	HAV
13	ALF5	1	F	668	171	6.38	II	Death	Acute liver failure	HAV
14	ALF6	24	M	964	762	3.3	II	Death	Acute liver failure	HBV
15	ALF7	31	F	314	562	2.1	II	Survival	Acute liver failure	HBV
16	ALF8	17	M	200	120	1.7	II	Survival	Acute liver failure	HAV

Legend: HS: healthy subjects; ALF: acute liver failure patients; HAV: hepatitis A virus; HBV: hepatitis B virus; F: female; M: male; NA: not applicable.

**Table 2:** Viral load obtained from plasma samples and HepG2 cell culture after ALF plasma exposure before and after heat inactivation.

Viral hepatitis	Viral load on plasma samples	Viral load on plasma samples (heat-inactivated)	Viral load on supernatant from HepG2 cells	Viral load on supernatant from HepG2 cells (heat-inactivated)	Plasma samples vs. plasma samples (heat-inactivated) (*p* value)	Viral load on supernatant from HepG2 cells vs. viral load on supernatant from HepG2 cells (heat-inactivated) (*p* value)
A	3.27 ± 0.43	2.58 ± 0.53	3.24 ± 0.81	2.48 ± 0.48	0.062	0.250
B	3.97 ± 0.32	3.73 ± 0.45	3.64 ± 0.32	3.28 ± 0.41	0.120	0.094

Legend: viral load data was expressed as log_10_ copies/mL and mean ± SE.

## Data Availability

The data used to support the findings of this study are included within the article.
